# Optimised Fermentation Production of Radiolabelled Ochratoxin A by *Aspergillus ochraceus* with Maximum ^14^C in the Pentaketide Moiety for Exploring Its Rat Renal Toxicology

**DOI:** 10.3390/toxins16010008

**Published:** 2023-12-22

**Authors:** Peter Mantle

**Affiliations:** Centre for Environmental Policy, Imperial College London, London SW7 2AZ, UK; p.mantle@imperial.ac.uk

**Keywords:** radioactive biosynthesis, optimised kinetics, wheat substrate, shaken culture, DNA adducts, OTA carcinogenesis

## Abstract

In the context of the mysterious Balkan endemic nephropathy of the 1900s, and the discovery in the 1960s of the potent mycotoxin ochratoxin A, experimental research projects sought to explore any inter-relationship. Experimental lifetime administration of the toxin to male rats had revealed renal DNA adducts with the toxin, correlated with renal tumours, confirmation of which required molecular evidence. Consequently, production of ^14^C-ochratoxin A of a high specific radioactivity was required, practical biosynthetic detail of which had not previously been published. A fermentation study of *Aspergillus ochraceous* was carried out during 2002 for a European project, to select for the production of high-quality ^14^C-ochratoxin A, necessarily exploring for the maximum diversion of ^14^C-sodium acetate into the pentaketide portion of mycotoxin. Experimentation necessarily had to optimise the competitive context of fungal growth dynamics and addition of the biosynthetic precursor in the early days of shaken-flask fermentation before adding the radiolabelled precursor. From optimal fermentation, 50 mg of the ^14^C ochratoxin A was supplied within a European project for DNA adduct experimentation, but that proved negative as subsequently published. Experimental description of the radiolabelled ochratoxin A production was later made in a doctoral thesis, but is first publicised here. Further review of the literature reveals an explanation for the published failure to confirm rat DNA/ochratoxin A adduct formation, for which further experimentation is now recommended.

## 1. Introduction

Ochratoxin is one of the first mycotoxins, discovered in South Africa from *Aspergillus ochraceus* [1]. It was without any specific toxic application but was soon recognised also as a Penicillium metabolite responsible for a major chronic toxic problem in the Danish bacon industry from previously unrecognised seasonal problems from storage of local barley crops [2]. Further extensive pharmacological study [3] in the USA required pure toxin which was provided for rats from *A. ochraceus* fermentation to demonstrate male rat carcinoma. More recent lifetime rat studies in London [4] also required the toxin from local fermentation of an Australian *A*. *ochraceus* [5]; commercial OTA was expensive (c £17,000 per gram).

Potential genotoxic properties of ochratoxin A (OTA) might play an important role in the mechanisms of OTA-induced toxicity and carcinogenicity in rats. Although there are reports indicating that OTA causes DNA damage [6,7], the status of the mycotoxin in respect to the formation of covalent DNA adducts in the kidney of laboratory animals is still unclear. Covalent binding of chemicals to DNA had been recognised as an important trigger of the multistage process that leads to carcinoma [8]. Resolving the question of whether a compound forms covalent adducts with DNA ultimately finds further application in human risk assessment.

However, if covalent DNA adducts are proved to be evident, the causal substance is classed as more hazardous, leading to more stringent exposure limits. Evidence has been presented for the presence [9,10,11] and also for the absence [12,13] of covalent OTA-generated DNA adducts. Around the turn of the millennium, consistent claims for the formation of covalent OTA-generated DNA adducts in the kidney were made only by a cluster of French and Tunisian laboratories [14,15,16], and it became evident that demonstration of these adducts appeared to depend primarily on the rigour of experimental methodology of 32p-postlabelling. Moreover, the precise nature and chemical composition of these DNA adducts are still matters of much controversy. Not surprisingly, an increasing necessity has been felt by advisory and regulatory bodies to conclusively resolve this matter and, if their occurrence and structure gets confirmed, to elucidate the role of OTA-generated DNA adducts in carcinogenesis. Detecting adducts in tumours does not necessarily imply causation; the tissue is relatively disorganised and places metabolic demand on circulating blood.

Hence, a European collaborative study was designed with colleagues at Würzburg University, Germany, in an attempt, ultimately, to answer the question whether OTA forms covalent DNA adducts in rat kidneys. This study was an integral part of a multidisciplinary research project entitled ‘Mechanisms of OTA-induced carcinogenicity as a basis for an improved risk assessment’, funded by the Fifth RTD Framework Program of the European Union (Contract No. QLK1-CT-2001-011614). An early part of the broad study described here was concerned with the biosynthesis of ^14^C-OTA, labelled in the isocoumarin moiety of the molecule to a high specific activity [17] to facilitate experimental assessment of DNA adducts. This ^14^C-OTA was destined for collaborative rat experiments to explore DNA adduction.

The use of highly sensitive accelerator mass spectrometry analysis (AC/MS) was planned to investigate whether OTA as a whole or as its excretory metabolite OTalpha could bind to DNA in rat kidneys. This method might allow detection of as little as one DNA adduct/10° nucleotides.

Contrary to 32p-postlabelling, detection of OTA-DNA adducts by AC/MS requires administration of highly radiolabelled OTA to experimental animals prior to analysis of tissue DNA. Labelling natural products such as fungal metabolites sufficiently for experimental purposes poses a challenge in itself but was developed to yield milligram quantities of OTA, labelled radioisotopically in the isocoumarin moiety to a high specific activity, by optimising exploitation of a natural producer of OTA, *A. ochraceus.*

The principle of shaken solid substrate fermentation was first devised for aflatoxins [18] and later applied to OTA production on a large scale [19]. However, the technique for the production of OTA by *A. ochraceus* under laboratory conditions has been refined for facilitating high yield and using efficient extraction and purification methods. Shredded wheat (SW), as commercially available as a standard breakfast cereal, has been shown to be an excellent substrate for high-yield OTA biosynthesis [20]. In shaken solid substrate fermentation on shredded wheat over 14 days, the *A. ochraceus* strain D2306, originally isolated from Australian soya meal [21], produces OTA in very high yield of up to 10 mg/g substrate [5]. OTA from this strain has been used in several toxicological studies in pigs and poultry [22,23].

**Figure 1:**
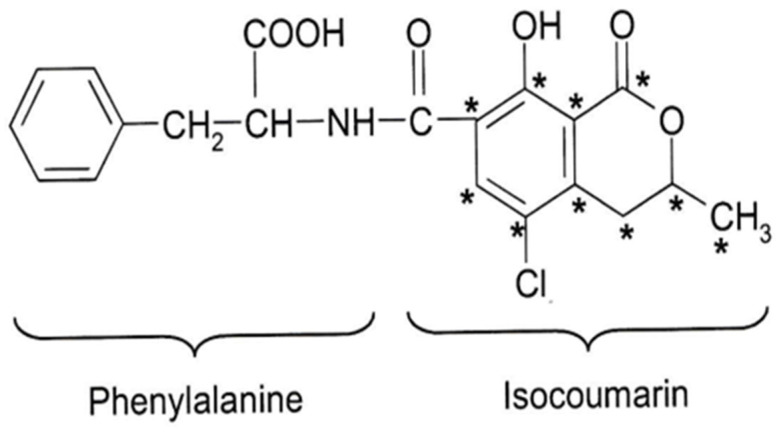
OTA labelled ^14^C in the isocoumarin moiety by [1,2-^14^C]-acetate in an *A. ochraceus* fermentation leads to incorporation of label in positions marked with *, except for the peptide bond carbon, derived from methionine.

For the biosynthesis of ^14^C-labelled OTA with high specific activity, a unique experimental protocol is vital and expected to be different from that particularly aimed at high-yield production of OTA. The challenge was to find the optimal time point during the early part of a fermentation with a high OTA production rate for giving a pulse of radiolabelled acetate, so that OTA’s isocoumarin moiety could be labelled to the highest possible specific activity commensurate with producing a sufficient amount of that product for subsequent animal experiments. Thus, it was essential to add the labelled precursor when the isocoumarin biosynthesis commenced and when virtually no unlabelled OTA had yet accumulated in the fermentation. Ideally, a maximum proportion of the labelled precursor should be used biosynthetically by the fungus for the ochratoxin pathway rather than for competing metabolic OTA labelled ^14^C in the isocoumarin moiety by [1,2-^14^C]-acetate in an *A. ochraceus* fermentation. This leads to the incorporation of label (Figure 1) in positions marked with *, except for the peptide bond carbon derived from methionine pathways. It was equally crucial to terminate the fermentation process before too much unlabelled OTA had been formed to dilute the overall specific radioactivity of the labelled mycotoxin. A minor amount of ochratoxin B (OTB), the des-chloro analogue of OTA, will always be a by-product of the fermentation and will be radiolabelled to a specific radioactivity of an order similar to that of OTA. However, it was desirable to achieve an OTA/OTB ratio that was as high as possible at the time of termination. In order to optimise all these conditions, a number of preliminary experiments were conducted to develop the protocol, including the selection of the optimum isolate *A. ochraceus*.

The specific aim here was to achieve a total yield of about 50 mg OTA with a minimum specific radioactivity of 0.25 mCi/mmol, which would enable the realisation of planned collaborative in vivo experiments designed to study the potential formation of OTA-generated direct or indirect covalent DNA adducts in rat kidneys.

## 2. Results

### 2.1. Ochratoxin Production by Three Isolates of A. ochraceus in Shaken Substrate

**Figure 2:**
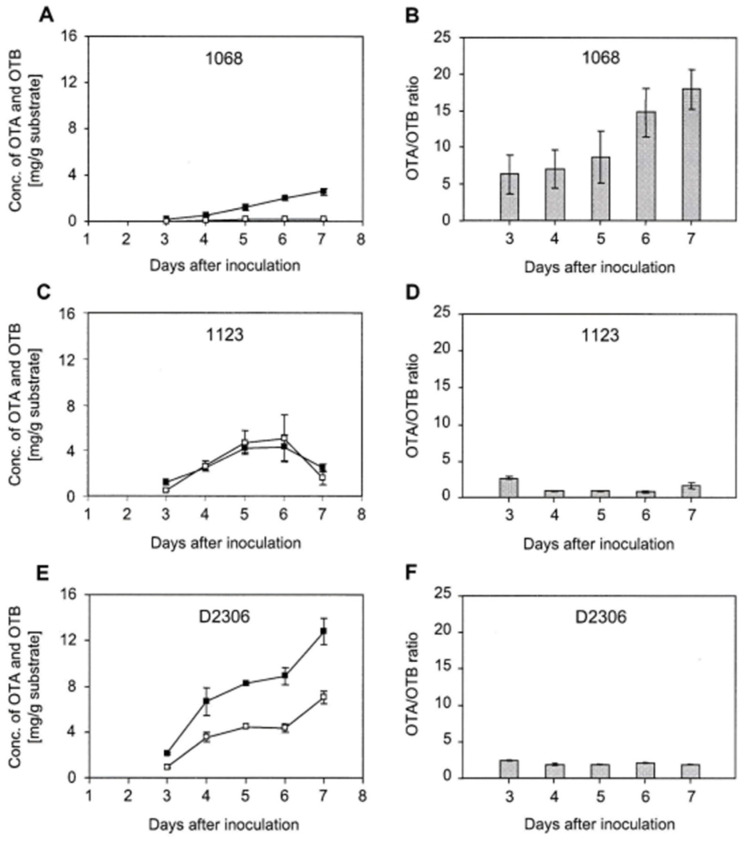
Ochratoxin production of different *A. ochraceus* isolates. Aliquots from shaken solid substrate fermentations were assayed at different time points and the yield of OTA (**A**) and OTB (**B**) was determined using HPLC as described in Methods. Concentrations of OTA and OTB per g of substrate and corresponding OTA/OTB ratios are shown for *A. ochraceus* isolates 1068 (**A**,**B**), 1123 (**C**,**D**) and D2306 (**E**,**F**). Experiments were performed in duplicate. All data shown are mean ± SD. The solid black blocks represents ochratoxin A (OTA). The hollow blocks represent ochratoxin B (OTB).

Growth experiments showed that both OTA yield and the OTA/OTB ratio were significantly dependent on the *A. ochraceus* isolate used under the larger-than-usual fermentation volume and rotation speed for ochratoxin biosynthesis (2 L flask with 160 g SW and 64 mL spore suspension incubated on an orbital shaker at 28 °C and 350 rpm). For preliminary experiments, all fermentations employing the various isolates were carried out in parallel, since such biosystems are very sensitive to marginal variations in the experimental setup. The first aliquot was assayed 3 days after inoculation, followed by daily sampling for up to 7 days. The *A. ochraceus* isolates 1068, 1123 and D2306 displayed very different OTA production curves (Figure 2).

OTA production of isolate 1068 commenced around day 3 post-inoculation and increased in a linear fashion during the period assayed, reaching a maximum concentration of 2.6 mg/g substrate (Figure 2A). The concentration of OTB stayed consistently low at around 0.1 mg/g substrate, resulting in a steady increase in the OTA/OTB ratio over time up to a value of 18 (Figure 2B).

In contrast, concentrations of OTA and OTB in fermentations of isolate 1123 were similar at all time points assayed, reaching a maximum of ca. 5 mg/g substrate by day 5 (Figure 2C). Consequently, the OTA/OTB ratio remained fairly constant over time at around a value of two (Figure 2D). Surprisingly, the concentration of both mycotoxins decreased on day 7 (Figure 2C). This could be illustrating some catabolism of the mycotoxins that would be undesirable for the purpose of synthesising radiolabelled OTA.

The highest yield of OTA was produced by isolate D2306, commencing prior to the first sampling on day 3 following inoculation. The concentration of OTA increased gradually over time, reaching a maximum of 12.8 mg/g substrate on day 7 (Figure 2E), and the OTA/OTB ratio remained around two (Figure 2F).

An OTA/OTB ratio > one is an important selection criterion for the most suitable organism for the particular purpose of producing radiolabelled OTA with a high specific activity. Thus, isolates 1068 and D2306 were the obvious candidates.

In contrast, isolate 1123 synthesised similar amounts of OTA and OTB, which would result in a less effective incorporation of ^14^C-labelled acetate into OTA.

Isolate 1068 achieved a significantly higher OTA/OTB ratio than isolate D2306, but it increased continuously over time. In contrast, D2306 displayed a constant ratio of both mycotoxins. The final concentration of OTA was significantly higher for D2306 (~12 mg/g substrate) compared to the two other isolates. This is consistent with previous results showing that isolate D2306 is a superior producer of OTA under a variety of laboratory conditions, achieving yields of up to 10 mg/g substrate. However, for the intended purpose of producing radiolabelled OTA with high specific radioactivity, especially at the early stage of the experiment, it is much more critical to ensure the reliable incorporation of ^14^C-labelled precursor.

It must be emphasised, however, that the yield of mycotoxins was similar in duplicate fermentations as illustrated by the error bars in Figure 2, but did vary across experiments not conducted in parallel, even under constant fermentation conditions. However, the OTA/OTB ratios were characteristic for each individual *A. ochraceus* isolate and were uncorrelated to the final yield of the mycotoxins. Thus, isolates D2306 and 1068 were selected for further studies.

### 2.2. Comparative OTA and OTB Production by A. ochraceus Isolates D2306 and 1068 in Shaken Solid Substrate Fermentation

**Figure 3:**
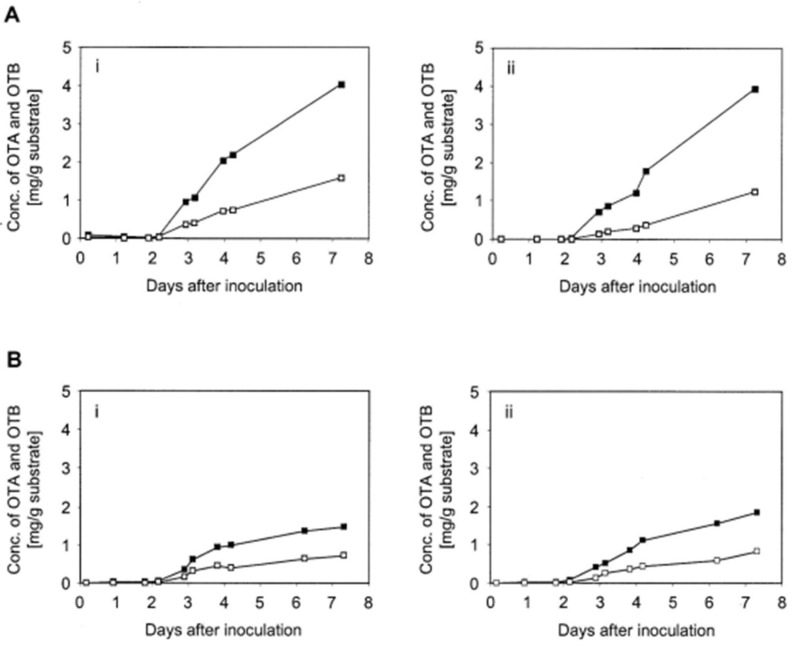
Early ochratoxin production of *A. ochraceus* isolate D2306. Two separate production experiments (**A**,**B**), each consisting of two replicate fermentation flasks (**i**,**ii**), displaying their yield profiles of OTA (black) and OTB (open). The solid black blocks represents ochratoxin A (OTA). The hollow blocks represent ochratoxin B (OTB).

It became apparent from the mycotoxin production curves that it was critical to know the onset of OTA synthesis in fungal fermentation to find an optimal time for the addition of radiolabel. OTA production by *A. ochraceus* isolates D2306 and 1068 was, therefore, monitored, especially in the early stages of shaken solid substrate fermentation. Under standard conditions, the onset of both OTA and OTB synthesis differed by about 24 h between both isolates. In D2306 fermentations, mycotoxin production commenced between days 2 and 3, irrespective of the subsequent rate of mycotoxin synthesis (Figure 3A,B). In both duplicates of fermentation A the OTA concentration reached around 1 mg/g substrate 3 days after inoculation, and it doubled to around 2 mg/g substrate after a further 24 h. However, another pair of D2306 fermentations yielded only about half that amount during the same period (Figure 3B), reaching an OTA concentration of around 0.5 mg/g substrate after 3 days and around 1 mg/g substrate after 4 days.

**Figure 4:**
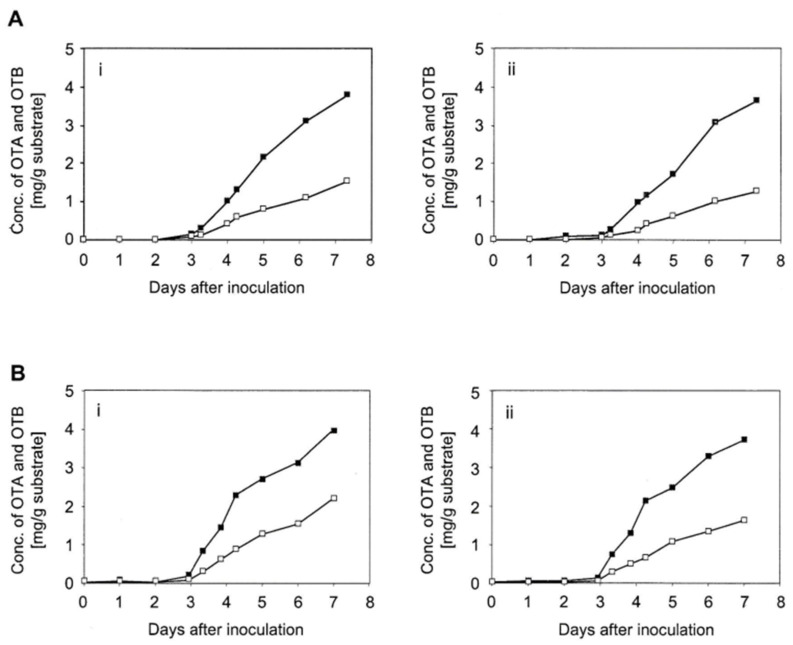
Early ochratoxin production of *A. ochraceus* isolate 1068 displayed in two experiments (**A**,**B**), each with two replicates (**i**,**ii**). The solid black blocks represents ochratoxin A (OTA). The hollow blocks represent ochratoxin B (OTB).

As illustrated in Figure 4, mycotoxin biosynthesis for isolate 1068 fermentations commenced consistently around day 3 post-inoculation, as was expected from previous experience. The increase in OTA concentration in the following 24 h reached up to 1.5 mg/g substrate and was comparable in both sets of duplicate fermentations. Five days following inoculation, the OTA concentration had reached around 2 mg/g substrate in both duplicate pairs. The consistent performance of isolate 1068 made it more suitable for the production of radiolabelled OTA in the required quantity and for a more efficient use of ^14^C acetate, making it the fungus of choice for the addition of the radiolabel at 72 h.

### 2.3. ^14^C-Acetate Feeding Experiment

For the next exploratory stage, a ^14^C-labelling fermentation was conducted in four conical 500 mL Erlenmeyer flasks containing 40 g sterilised SW, inoculated with 16 mL concentrated spore suspension of *A. ochraceus* isolate 1068. The flasks were thoroughly shaken to maximise distribution amongst the SW fragments, followed by incubation on an orbital shaker at 28 °C and 350 rpm with a 10 cm eccentric throw. An amount of 5 μCi [1,2-^14^C]-acetic acid sodium salt (>100 mCi/mmol) was dissolved in 8 mL of sterile H_2_O, and 2 mL of that solution was added to each flask 72 h post-inoculation. To ensure homogenous distribution of the radiolabel, the aqueous solution was sprayed as evenly as possible on to the substrate using a syringe with a fine metal needle. Each flask was shaken thoroughly prior to continued incubation under the same conditions. The experiment was terminated 24 h after the addition of the radiolabel, followed by partial purification of the fermentation product.

For each batch of crude extract, preliminary analyses by DAD-HPLC and scintillation counting of the relevant fractions revealed that both the yield of OTA and its specific activity were low. Thus, all four bicarbonate soluble extracts containing the acidic metabolites of the bio-fermentation were pooled prior to further purification by PLC.

Partially purified crude extracts from all four flasks were pooled and applied to a PLC plate at the origin, side-by-side with controls for OTA and OTB. The plate was developed and dried prior to its exposure to X-ray film for 4 days. The film was developed for 3 min using standard methods. Separation of the extract revealed four main bands which corresponded with the standards for OTB and OTA, respectively, that were visualised under UV light at 350 nm.

Separation of fractions 1 and 2 was poor, resulting in the lack of a distinct boundary between these two bands. However, the majority of the radiolabel was present in the area between the origin and the upper edge of band 2. Examination of the PLC plate under UV light at 350 nm revealed that bands 1 and 2 emitted blue fluorescence typical for OTA and OTB. Identification of the mycotoxin controls under UV light at 350 nm revealed that the OTB control had travelled to the bottom edge of band 1, whereas the OTA control was located in close proximity at the bottom edge of band 2. This confirms that the chromatographic separation was insufficient for obtaining two distinct bands for the fractions containing OTA and OTB.

A smear was visible across the plate, particularly proximal to the origin and near the solvent front, indicating that too much extract may have been applied to the origin. Another possible explanation for the inferior separation of compounds could be disturbance of the equilibrium of the solvent mixture in the chromatographic tank by leakage, allowing for evaporation of the solvent, which would result in a gradual change in the solvent composition. Nevertheless, the position of all compounds visible under UV light at 254 nm matched the bands and smears visualised in the autoradiograph.

**Figure 5:**
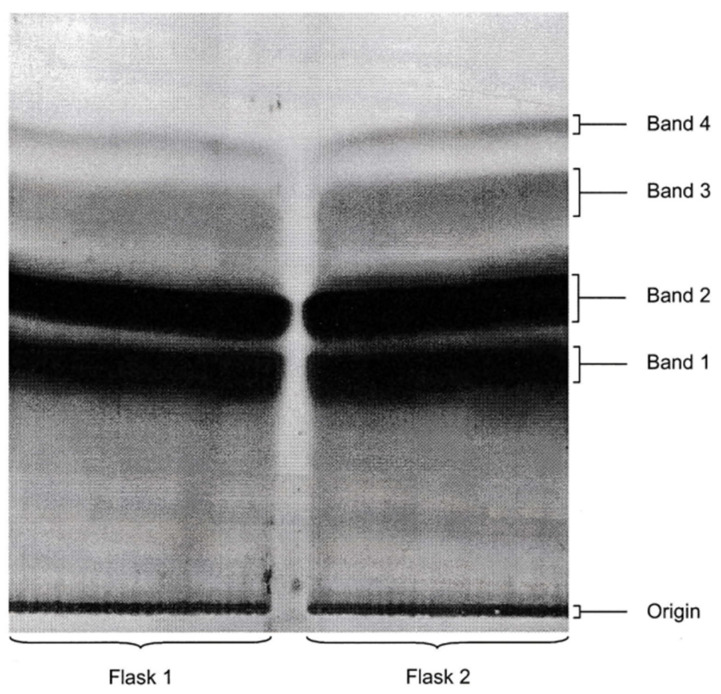
Second ^14^C-labelling experiment for ochratoxins: flasks 1 and 2. Autoradiograph of PLC plate following separation of bicarbonate-soluble extracts from two replicate fermentation flasks. Chromatography solvent: toluene/ethyl acetate/formic acid (5:4:1). Band 1, OTB; Band 2, OTA.

For a revised strategy for OTA production, four 500 mL fermentation flask cultures were given the precursor (5 mCi of 1,2-^14^C acetate in 8 mL water) after 72 h of shaken incubation. Each flask received 2 mL through a syringe with a very fine needle, sprayed in several small amounts between vigorous flask shaking. After 24 h, fermentation was terminated. Following extraction and chromatographic separation, autoradiography revealed products (Figure 5), identified by previous experience. Isolation of the OTA and OTB revealed, for example, 80 mg of OTA into which 1% of the ^14^C-acetate had been incorporated to give a specific radioactivity of 0.25 mCi/mmol.

## 3. Discussion

In the context of whether OTA has significant animal toxicity, particularly for generating renal malignancy, a key publication [14] describes renal analysis for evidence of OTA binding to molecular targets. Rats had been dosed three times per week for life. Several renal tumours occurred in males, but none in females. Male-exclusive association of OTA with renal genome was expressed, as a range of DNA adducts were revealed in a chromatographic display. Reproducability and significance of this could have been related to current opinions that, if a toxin failed to make DNA adducts, its perceived risk for human disease might be 10-fold reduced. Such was naturally a topic around the turn of the millennium. However, the cited study barely mentioned the extensive pharmacological experimentation in the USA [3] in the 1980s, which had established a major gender difference in rat susceptibility to a similar dosage of OTA, which had also complemented a similar but more extreme gender sensibility in mice [24]. Subsequently, at the turn of the millennium, renewed research concerning DNA adduction requested the provision of OTA radiolabelled to a high degree, as currently described. Published experimentation [25] failed to confirm DNA adduction, although a subsequent study [26] showed that a single oral dose of OTA resulted in the virtual disappearance of any OTalpha residue during a subsequent 3-day gap before a repeated dose cycle again showed the same pattern. Thus, the single ^14^C-OTA gavage dosing of rats for the adduction study [25], followed by 3 days before analysis for renal DNA adducts, could allow major excretion of the radiolabelled OTalpha before the kidney tissue was analysed and a negative conclusion was reached. That study also notably failed to acknowledge the dedicated experimentation to create the ^14^C OTA, presently described from the cited thesis [17]. If the presently described ^14^C-OTA still exists, male rats could even be maintained for two or more weeks on a higher-than-usual OTA diet before their kidneys are used for critical analysis for DNA adducts, supported concurrently by analytical proof of a significant circulating vascular concentration of OTA. The labelled OTA could even be given by oral gavage as two separate additions on the day before adduct analysis. For OTA, inclusion of female rats might show a contrasting difference in DNA adduction after circulating OTA reached a maximum concentration in well-matched dosing, conveniently and tolerantly assured by daily dietary OTA at about 300 μg/kg b.wt. and potentially on a trajectory leading ultimately to revealing nearly 100% renal tumours in males.

EFSA reported in 2006 that there was still uncertainty about OTA adducts, and more recently [27] reported that the formation of specific OTA-DNA adducts remained controversial. Possibly, some unused ^14^C OTA remains and could be used for a well-designed experiment. Meanwhile, an independent experience of rat DNA adduct analysis in the Toulouse laboratory had also revealed a positive signal for another mycotoxin from *Penicillium polonicum* which also produces apoptosis-like pathology in rat kidneys [28,29].

The purpose so far has been to report the general principles underlying the historic production of ^14^C OTA. Recognising the gender difference in rat sensitivity to OTA could relate uniquely to that concerning small proteins (approximately 20 kiloDaltons) in the circulating blood of male rats and mice, associated with their fundamental role in sexuality [30]. This had been exploited by an OTA primary antibody diverted from its role in selective isolation columns (R-Biopharm Rhone Ltd., Glasgow, UK). Only temporarily available, its OTA binding was shown to mimic the binding of OTA to sexuality-significant small molecules in male rat blood [31]. Such small proteins could also pass OTA naturally through renal cortical corpuscles for excretion, after which their proximity to nephron epithelia could also allow for the transfer of OTA, with both toxic consequence and its cleavage to release the valuable aromatic amino acid phenylalanine. The other product, OTalpha, is consequently released for excretion through the rest of each nephron.

The original adduct study [14] exposed rats to OTA for a lifetime. An attempt to verify adducts during a short exposure might be a challenge, because maximum circulating OTA takes about a month to achieve by continuous daily exposure [32]. Its circulating concentration can be greater in females [32], presumably because of no male sexuality peptides exist in female rat blood. Historically, vascular OTA was impossible to monitor in the 1980s NTP study [3] and in the early adduct experiment [14], and there would have been no basis to explain any much higher OTA value in females. The more recent demonstration [31] that male sexuality proteins can be important in augmenting OTA flow from blood into the upper nephrons can simply explain the gender difference. Thus, for any repeat of attempts to verify whether DNA adduction occurs in male rats, at least two weeks of dietary OTA exposure at a high dose may be necessary before also giving ^14^C-OTA for at least two days would likely create a reasonable basis for adduct analysis. In any case, it has already been suggested that OTA/rat renal carcinoma may simply reflect those developing naturally in the Eker rat [33]. Further, there is also still a need to determine whether OTA/rat tumours are really predictive for humans.

Further from the 2015 study in Turkey [32], commencing maintenance of male and female rats of a similar age on the same dietary OTA (300 μg/kg food) for several weeks doubled the accumulated blood concentration of the toxin. This could be consistent with male vascular sexuality proteins assisting with OTA’s vascular excretion through enhanced release into and through kidney nephrons. The origin of these small proteins during puberty was explored [34] within the context of some potentially being involved directly in enhancing the access of OTA to nephron epithelia for its expression of toxicity. Sadly, the tragic death of my colleague Judit Nagy curtailed further such exploration at Imperial College London.

## 4. Materials and Methods

### 4.1. Reagents

Sigma-Aldrich Company Ltd. (Poole, Dorset, UK) supplied acetic acid, phosphoric acid (H_3_PO_4_), formic acid, potassium chloride (KCI), sodium chloride (NaCI), sodium hydrogen carbonate (NaHCO_3_), scintillation fluid and Triton X-20.

Other companies supplied the following: [1,2-^14^C]-acetic acid sodium salt (>100 mCi/mmol; Hartmann Analytic, Braunschweig, Germany), acetonitrile, ethyl acetate, methanol, propan-2-ol, toluene (BDH Laboratory Supplies, Poole, Dorset, UK), potato dextrose agar (PDA; Difco, Becton Dickinson, Cowley, Oxfordshire, UK), Shredded Wheat (Cereal Partners UK, Welwyn Garden City, Hertfordshire, UK), glass preparative layer chromatography (PLC) plates (Sil 60G-200/UV254, Camlab, Cambridge, Cambridgeshire, UK), X-ray film, D19 Developer (Kodak, Rochester, NY, USA), Ilfostop and Hypam Rapid Fixer, (Ilford, Mobberley, Cheshire, UK).

### 4.2. A. ochraceus

Maintenance and cultivation of *A. ochraceus* isolate D2306 were as used previously [20], and isolates 1068 and 1123 were maintained on potato dextrose agar (PDA) slopes. PDA slopes consisted of 10 mL PDA medium, autoclaved in 30 mL universal bottles. For maintaining a maximum yield of OTA, several sub-cultures of *A. ochraceus* were incubated for up to 2 weeks, until the fungus was sporulating freely and the agar had become blue fluorescent under UV light at 350 nm due to the presence of OTA. The slopes showing the brightest fluorescence were stored at 4 °C as primary sources of inoculum for shaken solid substrate fermentation. Spores from the slopes were used to inoculate 20 g of loosely broken shredded wheat biscuits (SW), first autoclaved in a 250 mL conical flask and then thoroughly mixed with 8 mL sterile H_2_O. These SW cultures were incubated stationary until heavy sporulation for up to 3 weeks at 28 °C. They were stored at 4 °C until being used as an inoculating source for shaken solid substrate fermentations for OTA production.

### 4.3. Biosynthesis of Ochratoxin A

Standardised OTA production was performed by growing *A. ochraceus* in shaken substrate fermentations. A spore suspension was prepared by homogenising a heavily sporulating stationary SW culture in a 42 mM KCI solution containing 1% Triton X-20. Addition of potassium chloride in the inoculum of all fermentations ensured adequate chlorine availability for maximum OTA production. The natural chlorine content of SW might otherwise limit OTA production to about 10 mg/g substrate. 40 g SW, autoclaved in conical 500 mL Erlenmeyer flasks with foam bungs, was inoculated with 16 mL of spore suspension, resulting in an initial moisture content of 40%. Alternatively, 64 mL of spore suspension was added to 160 g SW, autoclaved in 2 L Erlenmeyer flasks. Flasks were thoroughly shaken to equilibrate distribution amongst the SW fragments and the cultures were incubated on an orbital shaker (200 or 350 rpm with a 10 cm eccentric throw) for up to 22 days at 28 °C.

Over this period, cultures gradually became yellow–brown and eventually darkened to a chocolate colour as secondary fungal pigments were produced. The substrate fragmented further to particles of only a few millimetres in length and bore no spores on account of constant friction. The only sporulation occurred above the level of substrate rotation where metabolic water condensed early in the fermentation. The final fermentation product was composed of separate SW fragments that had been fully colonised internally by fungal hyphae and from which most nutrients had been absorbed through assimilation into fungal biomass.

### 4.4. Biosynthesis of ^14^C-Radiolabelled Ochratoxin A

The method for biosynthesis of ^14^C-radiolabelled OTA is based on the same principle as that for the production of unlabelled OTA and also employs the shaken solid substrate fermentation on SW. Preliminary experiments were conducted to find optimal conditions in order to produce OTA of high specific radioactivity. First, OTA production curves of different sub-isolates of *A. ochraceus* were determined as follows. SW, autoclaved in Erlenmeyer flasks with foam bungs, was inoculated with a spore suspension that was prepared as described earlier. This was carried out on two scales: 64 mL spore suspension was added to 2 L flasks with 160 g SW, and 16 mL of spore suspension was added to 500 mL flasks containing 40 g SW. The flasks were thoroughly shaken and incubated on an orbital shaker (350 rpm with a 10 cm eccentric throw) for 7 days at 28 °C. Small samples were taken from the fermentation under sterile conditions at several time points, acidic metabolites were extracted and analysed for their OTA and OTB content by high pressure liquid chromatography (HPLC), as described in the methods.

Once the most suitable culture of *A. ochraceus* had been selected, preliminary experiments were carried out to determine the optimum time point at which the labelled precursor had to be added to the culture, and also when the fermentation had to be terminated to achieve the most effective incorporation of the radiolabelled precursor and to yield OTA of the highest possible specific radioactivity.

Briefly, SW was inoculated with spore suspension of *A. ochraceus* in Erlenmeyer flasks with foam bungs and incubated on an orbital shaker at 28 °C. An amount of 5 μCi of [1,2-^14^C]-acetic acid sodium salt, dissolved in 2 mL sterile H_2_O, was added to each flask. To ensure homogenous distribution of the label, the aqueous solution was sprayed evenly onto the substrate using a syringe with a fine metal needle. Each flask was shaken thoroughly before returning it to the orbital shaker to continue incubation in the same conditions.

From each fermentation, small samples were taken at several time points, and their acidic metabolites were extracted. A total of 20 μL of the concentrated extract was analysed and separated using HPLC. The OTA and OTB content and their specific activity were analysed using scintillation counting. All preliminary experiments were carried out in duplicate.

Once the optimal parameters had been chosen, the actual labelling synthesis was carried out. In order to monitor OTA biosynthesis, a pair of identical fermentations was set up, one of which was used for continuous sampling and rapid analysis. The pairs were staggered by 24 h. For the final production, four 500 mL flasks with 40 g SW each were inoculated with 16 mL of spore suspension and incubated on an orbital shaker at 300 rpm with a 10 cm eccentric throw at 28 °C. Then, 72 h after inoculation, 1.25 mCi of [1,2-^14^C]-acetic acid sodium salt, dissolved in 2 mL H_2_O, was added to each flask, amounting to a total radioactivity of 5 mCi. Fermentations were returned to the orbital shaker for 24 h, after which biosynthesis was terminated by the addition of 0.01 M H_3_PO_4_ in ethyl acetate (1:9; *v*/*v*). Product isolation and purification was carried out as described below. Radiolabelled material was always handled, stored and disposed of in accordance with the College and University rules and regulations.

### 4.5. Extraction and Purification of ^14^C Ochratoxin A

At the end of the incubation period, the fermentation matrix was acidified to promote efficient extraction by mixing thoroughly with 0.01 M H_3_PO_4_ in ethyl acetate (1:9; *v/v*) and soaking the mixture overnight in sufficient volume to cover the matrix at room temperature in a closed glass vessel. The mixture was then filtered, residue was washed with more solvent, and the combined filtrate was partitioned twice against half the volume of 3% (*w*/*v*) aqueous NaHCO_3_. The bicarbonate phase, containing acidic metabolites, mainly the ochratoxins, was separated, acidified with HCl and extracted repeatedly with a third volume of ethyl acetate. The organic phase was separated and rotary evaporated to dryness. The residue containing the bicarbonate soluble compounds was then dissolved in a minimal volume of methanol, usually 1.5 mL, prior to preparative layer chromatography (PLC) or stored at 4 °C until analysis by HPLC. PLC was carried out on 20 × 20 cm glass plates, pre-coated with a 2 mm layer of silica gel containing a fluorescent indicator that emits UV light at 254 nm. Partially purified crude fermentation extracts containing ochratoxins were applied as a continuous band 2 cm from the base of the plate and thoroughly dried. The applied amount varied depending on the viscosity of the material. Standards of OTA and OTB were also applied to the plate. The plates were developed with toluene/ethyl acetate/formic acid, 5:4:1 (*v*/*v*/*v*) in a glass tank until the solvent front nearly reached the top edge of the plate.

Plates were air-dried overnight and aterial was eluted with propan-2-ol. The mixture was filtered through glass sinter funnel. The filtrates of three consecutive elutions were combined and dried by rotary evaporation. The residue was re-dissolved in a known volume of methanol and centrifuged at 14,000 rpm for 5 min to remove any fine silica particles. The supernatant was transferred to a new tube and stored at 4 °C until further use. All purified samples were assessed for quality and quantity prior to their use in further experiments.

Ultimately, the final fermentation yielded 80 mg of ^14^C-OTA and 30 mg of ^14^C-OTB, most of which were made available for DNA adduct studies [25] cited widely, for example, in [35,36,37,38,39,40,41,42,43].

## Data Availability

The data presented in this study are available on request from the corresponding author.
